# Supplementary material for: “The phone number tells us good things we didn’t know before.” Use of interactive voice response calling for improving knowledge and uptake of family planning methods among Maasai in Tanzania
Source: PLOS Digit Health. 2023 May 19;2(5):e0000254. doi: 10.1371/journal.pdig.0000254 (PMC10198512; doi:10.1371/journal.pdig.0000254)
Supplement: S1 Appendix — (PDF) [file pdig.0000254.s001.pdf]

Survey

Study ID ________________________

Husband ID ________________________

Screening

Initials ________________________
(3 digits)

Screening date ________________________
(dd/mm/yy)

Sex
- ◯ Male
- ◯ Female

Inclusion criteria
- ☐ Husband or wife of Maasai couple
- ☐ Age between 12-65 for men/12-50 for women
- ☐ Willing to receive/user IVRC
- ☐ Able to understand and willing to sign the, informed consent document
- ☐ In possession of a mobile phone or willing to use a provided mobile phone
- ☐ Living in Esilalei

(Check if yes)

EXclusion criteria
- ☐ Women of non-childbearing age
- ☐ Women who have had a tubal ligation procedure
- ☐ Women who are known to be infertile

(check if no)

Demographics

DEM1. Sex
- ◯ Female
- ◯ Male

DEM2. How old are you? (include approximation) ________________________

DEM2. If she knows date of birth ________________________
(dd/mm/yy)

DEM2. How old are you? How old on the last birthday how old she was ________________________
(yrs)

DEM2. How old are you? Time event(season) ________________________

DEM3. Where do you live? Region ________________________

DEM3. Where do you live? District ________________________

DEM3. Where do you live? Ward ________________________

DEM4. Place of birth? Region ________________________

DEM4. Place of birth? District ________________________

DEM4. Place of birth? Ward ________________________

DEM5. What language do you speak most often at home?

- ◯ Swahili
- ◯ Maa
- ◯ English
- ◯ Other

DEM5. What language do you speak most often at home? Other specify

____
(specify if other checked)

DEM6. What is your religion?

- ◯ Christian
- ◯ Muslim
- ◯ No religion
- ◯ Other religion

DEM6. What is your Christian? Other Christian religion specify

____

DEM6. What is your religion? Other religion specify

____

DEM7. What is the highest level of school that you completed?

- ◯ No formal education
- ◯ Standard 1
- ◯ Standard 2
- ◯ Standard 3
- ◯ Standard 4
- ◯ Standard 5
- ◯ Standard 6
- ◯ Standard 7
- ◯ Secondary education (specify level attained)
- ◯ Higher education (specify field)

(Secondary education specify level below, higher education specify field below)

DEM7. What is the highest level of school that you completed? Secondary education , specify level attained

____

DEM7. What is the highest level of school that you completed? Higher education specify field

____

DEM8. Can you read and count in swahili?(literary rate)

- ◯ No
- ◯ Yes

Socio-Economic Status

SES1. Does the house where you live have electricity/sola power?

- ◯ No
- ◯ Yes

SES2. Does the house where you live have piped water inside/rain catchment/water filters

- ◯ No
- ◯ Yes

SES3. Do you own a cell phone and its working?

- ◯ No
- ◯ Yes

SES4. Are you currently working in any sector where you receive a regular monthly salary?

- ◯ No
- ◯ Yes

(if no (skin to SES 6))

SES5. If employed: what type of work do you do?

____
(skip to SES7)

SES6. If not employed: If you are not formally employed, what do you do t o earn an income?	◯ No income earning activities<br>◯ Horticulture<br>◯ Pastoralist<br>◯ Petty trader<br>◯ other, specify
If pastoralist: number of cattle's	◯ 25-50<br>◯ 50-75<br>◯ 75-100<br>◯ 100-125<br>◯ 125-150<br>◯ >150
SES6. If not employed: If you are not formally employed, what do you do t o earn an income? Other specify	________________________________
SES7: If any work: How much money do you earn per month from these activities?(on average)	________________________________<br>(Tsh.)
SES8. Do you have an account in a bank or other financial institution that you yourself use?	◯ No<br>◯ Yes
SES9. Do you use your mobile phone for any financial transactions?	◯ No<br>◯ Yes
SES10. Have you ever used the internet?	◯ No<br>◯ Yes<br>(No, skip to SES13)
SES11. In the last 12 months, have you used the internet?	◯ No<br>◯ Yes
SES12. During the last one month, how often did you use the internet?	◯ Not at all<br>◯ Almost every day<br>◯ At least once a week<br>◯ Less than once a week
SES13. Are you covered by any health insurance?	◯ No<br>◯ Yes<br>(No, skip to PAR1)
SES14. What type of health insurance are you covered by?	◯ CHBi<br>◯ NHIF<br>◯ Other, specify
SES14. What type of health insurance are you covered by? Other specify	________________________________

Maternity

MAR1. Have you ever had children?	◯ No<br>◯ Yes<br>(No, skip to KCM1)
MAR2. Do you have any children that are now living with you?	◯ No<br>◯ Yes
MAR3. Do you have any children that are alive but do not live with you?	◯ No<br>◯ Yes

MAR4. Have you ever had children who were born alive but later died?
◯ No
◯ Yes

MAR5.Did all the children have the same biological father?
◯ No
◯ Yes

MAR6. How old were you when you first child was born?
____
(2 digits)

MAR7. How old is your youngest child?
____
(2 digits)

MAR8. Have you ever had a pregnancy that miscarried, was aborted, or ended in stillbirth?
◯ No
◯ Yes

MAR9. When did your last menstrual period start; if remember date
____
(dd/mm/yy)

MAR9. When did your last menstrual period start;.... Days ago
____

MAR9. When did your last menstrual period start; ....Weeks ago
____

MAR9. When did your last menstrual period start; Description of the moon
____

MAR9: No date of last menstrual period
☐ Unknown
☐ Don't remember

MAR10. Now i would like to ask you about a woman's risk of pregnancy. From one menstrual period to next, are there certain days when a woman is more likely to become pregnant when she has sexual relations?
◯ No
◯ Yes
(No, skip to MAR12)

MAR11. If YES: What description best this time?
◯ Just before her period begins
◯ During her period
◯ Right after her period has ended
◯ Halfway between two periods
◯ Other, specify

MAR11. If YES: What description best this time? Other specify
____

MAR12. After the birth of child, can a woman become pregnant before her menstrual period has returned?
◯ No
◯ Yes

Knowledge on Contraceptive Methods

KCM1. Women can have an operation to avoid having any more children?
◯ No
◯ Yes

KCM2. Men can have an operation to avoid having any more children?
◯ No
◯ Yes

KCM3. Women can have a loop or coil placed inside them by a doctor or a nurse which can prevent pregnancy
◯ No
◯ Yes

KCM4. Women can have an injection by a health provider that stops them from becoming pregnant for one or more months
◯ No
◯ Yes

KCM5. Women can have one or more small rods placed in their upper arm by a doctor or nurse which can prevent pregnancy for one or more years
◯ No
◯ Yes

KCM6. Women can take a pill every day to avoid becoming pregnant
◯ No
◯ Yes

KCM7. Men can put a rubber sheath on their penis before sexual intercourse
◯ No
◯ Yes

KCM8. As an emergency measure, within three days after they have unprotected sexual intercourse, women can take special pills to prevent pregnancy
◯ No
◯ Yes

KCM9. A woman uses a string of colored beads to know the days she can get pregnant. On the days she can get pregnant, partner will use condom or does not have sexual intercourse
◯ No
◯ Yes

KCM10. Up to six months after childbirth, before, the menstrual period has returned, women can use a method to prevent pregnancy which requiring frequent breastfeeding day and night
◯ No
◯ Yes

KCM11. To avoid pregnancy, women do not have sexual intercourse on days of the month they think they can get pregnant
◯ No
◯ Yes

KCM12. Men can be careful and pull out before climax
◯ No
◯ Yes

KCM13. Have you heard of any other ways or methods that women or men can use to avoid pregnancy
◯ Yes
◯ No

If yes, mention ______________________________

KCM14. In the last months have you heard about family on the radio?
◯ No
◯ Yes

KCM15. Seen anything on the television about family planning?
◯ No
◯ Yes

KCM16. Read about family planning in a newspaper or magazine?
◯ No
◯ Yes

KCM17. Received a voice or text message about family planning on mobile phone
◯ No
◯ Yes

KCM18. Been in a seminar about family planning?
◯ No
◯ Yes

KCM19. In the last few months, have you discussed family planning with a health professional?
◯ No
◯ Yes

KCM20. Contraception is a woman's concern and a man should not have to worry about it?
◯ Yes
◯ No

KCM21: If yes, how is it?
- ◯ Before her day have started
- ◯ During her days
- ◯ After finishing her days
- ◯ In between her days
- ◯ Different

KCM22. After having a child, a woman can get pregnant before her days are coming back?
- ◯ Yes
- ◯ No

KCM23. Contraception is a woman's concern and a man should not have to worry about it?
- ◯ Disagree a lot
- ◯ Disagree a little
- ◯ Agree a little
- ◯ Agree a lot

KCM24. Women who use contraception may become promiscuous?
- ◯ Disagree a lot
- ◯ Disagree a little
- ◯ Agree a little
- ◯ Agree a lot

Relationship Status and History

RSH1. What best describes your current relationship status?
- ◯ Married
- ◯ Single, not in a relationship(skip to RSH7)
- ◯ In relationship, but not married
- ◯ Separated from spouse/divorced(skip to RSH8)
- ◯ Widow (skip to (RSH8)

RSH2. Do you currently live with your partner/husband?
- ◯ Yes, all the time
- ◯ Yes, sometimes
- ◯ No

RSH3. Does your husband has more than one wife?
- ◯ No
- ◯ Yes

(No, skip to RSH8)

RSH4. Including yourself, in total, how many wives or live in partners does he have?

____
(2 digits)

RSH5. Are you the ________wife/live-in partner
- ◯ First
- ◯ Second
- ◯ Third
- ◯ Forth
- ◯ Other, specify

RSH5. Are you the ________wife/live-in partner, other specify

____

RSH6. Are you living together with all co-wives/live-in partners in one house/boma?
- ◯ No
- ◯ Yes

RSH7. Have you ever been married or lived together with a man?
- ◯ No
- ◯ Yes

(No, skip to SAC1)

RSH8. Have you been married or lived with a man more than once?
- ◯ No
- ◯ Yes

RSH9. How old were when you first started living with a man?

____
(2 digits)

Sexual activity and contraceptives

SAC1. How old were you when you had sexual intercourse for the very first time?

____
(2 digits)

SAC2. When was the last time you had sexual intercourse?

- ◯ Day ago
- ◯ Weeks ago
- ◯ Months ago
- ◯ Years ago

SAC3. Sex with the last sexual partner, Was a condom used?

- ◯ No
- ◯ Yes

SAC4. Was a condom used every time you had sexual intercourse with the last person?

- ◯ No
- ◯ Yes

SAC5. What was your relationship to the person you had sex with last?

- ◯ Husband
- ◯ Partner
- ◯ Boyfriend
- ◯ Others, specify

SAC5. What was your relationship to the person you had sex with last? Other specify

____

SAC6. How long ago did you first have sexual intercourse with this person? ____days ago

____

SAC6. How long ago did you first have sexual intercourse with this person? ____weeks ago

____

SAC6. How long ago did you first have sexual intercourse with this person? ____Months ago

____

SAC6. How long ago did you first have sexual intercourse with this person? ____Years ago

____

SAC7. How many times during the last___(refer to SAC6) did you have sexual contact with this person?

____

SAC8. How old is this person?

____

SAC9. Was this person circumcised?

- ◯ No
- ◯ Yes

SAC10. A part from this person, have you had sexual intercourse witha ny other person in the past 12 months?

- ◯ No
- ◯ Yes

(No, skip to SAC12)

SAC11. In total, with how many different people have you had sexual intercourse in the last 12 months

____

SAC12. In the last 12 months, were you being paid by anyone in exchange for having sexual intercourse?

- ◯ No
- ◯ Yes

SAC13. In the last 12 months, did you pay anyone in exchange for having sexual intercourse?

- ◯ No
- ◯ Yes

SAC14. In total, with how many different people have you had sexual intercourse in your lifetime?

____

SAC15. If SAC3 was YES, ask the following questions. If NO skip to SAC21
You told me that a condom was used the last time you had sex. What is the brand name of the condom used at that time?

____

SAC16. From where u obtain the condom the last time?

____

SAC17. In the past 12 months have you given any gift or other goods in order to have sex or become sexually involved with someone?

◯ Yes
◯ No

SAC18. The last time you had sex did you or your partner use any method other than a condom to avoid or prevent a pregnancy?

◯ No
◯ Yes
(No, skip to SAC21)

SAC19. What method did you or your partner use?

◯ Female/male sterilization
◯ IUD
◯ Injectables
◯ Implants
◯ Pill
◯ Male/female condom
◯ Emergency
◯ Standard days method
◯ Lactational amenorrhea method
◯ Rhythm method/withdrawal
◯ Abstain
◯ Other modern method
◯ Other traditional method

Other modern Mention

____

Other traditional mention

____

SAC20. If answer for SAC 19 IS 11 or 12, ask to specify

____

SAC21. Do you know of a place where you can obtain a method of family planning?

◯ No
◯ Yes
(Yes, mention below)

SAC21. Do you know of a place where you can obtain a method of family planning? Yes mention

____

Fertility preferences

FP1. Are you pregnant now?

◯ No
◯ Yes
(No, skip to FP7)

FP2. How many months pregnant are you now?

____

FP3. When you got pregnant, did you want to get pregnant at that time?

◯ No
◯ Yes
(yes, skip to FP9)

FP4. How much longer did you want to wait?

____

FP5. After the child your (wife/partner) are expecting now, would you like have another child?

◯ No
◯ Yes
(No, skip to FP9)

FP6. After the birth of the child you are expecting now, how long would you like to wait before the birth of another child?

____

FP7. If MAR1 was NO, skip to FP9.
Would you like to have another child?

◯ No
◯ Yes
(No, skip to FP9)

FP8. How long would you like to wait from now before the birth of another child?

____
(Years (if month are given, please calculate years by taking the number of months devided by 12))

FP9. If you could go back to the time you did not ahve any children and could choose exactly the number of children to have in your whole life, how many would that be?

____
(skip to HIV1)

FP10. From now how much longer do you want to wait before you become pregnant?

____
(Years (if month are given, please calculate years by taking the number of months devided by 12))

HIV/AIDS General Knowledge and Awareness

HIV1. Now i would like to talk about something else. Have you ever heard of HIV or AIDS?

◯ No
◯ Yes

HIV2. HIV is the virus that can lead to AIDS. Can a people reduce their chance of getting HIV by having just one uninfected sex partner who has no other sex partners?

◯ No
◯ Yes
◯ I don't know

HIV3. Can people reduce their chance of getting HIV by using a condom every time they have sex?

◯ No
◯ Yes
◯ I don't know

HIV4. Can HIV be transmitted from a mother to her baby? (circle/tick all that applies)

☐ During pregnancy
☐ During delivery
☐ By breastfeeding
☐ I don't know

Past Medical History on STI

PMHS1. During the last 12 months, have you had a disease which you got through sexual contact?

◯ No
◯ Yes

PMHS2. Sometimes women experience an abnormal discharge from their vagina. During the last 12 months, have had an abnormal discharge from your vagina?

◯ No
◯ Yes

PMHS3. Sometimes women have sore or ulcer near their vagina. During the last 12 months, have you had a sore or ulcer on or near your vagina?

◯ No
◯ Yes

PMHS4. Ask this question if client has answered YES to any of the above questions. If NO skip to SRPW1

The last time you had (this problem) did you seek any kind of advice or treatment?

◯ No
◯ Yes
(No, skip to SRPW1)

PMHS5. Where did you go?
(probe for more all possible places) __________________________________

Sexual relationship with partner/wife

SRPW1. If a husband knows his wife has a disease that he can get during sexual intercourse, is he justified in asking that they use condom when they have sex?

○ No
○ Yes

SRPW2. Is a husband justified in refusing to have sex with his wife when he knows she has sex with other men?

○ No
○ Yes

Circumcision

CIR1. Some women are circumcised, that is, the genitals have been cut. Are you circumcised?

○ NO
○ Yes
(No, end interview)

CIR2. How old were you got circumcised? __________________________________
(Years (if month are given, please calculate years by taking the number of months devided by 12))

CIR3. Who did the circumcision? __________________________________

CIR4. Where was it done? __________________________________

CIR5. How do you feel about being circumcised? __________________________________

Comments __________________________________
