# Supplementary material for: “The phone number tells us good things we didn’t know before.” Use of interactive voice response calling for improving knowledge and uptake of family planning methods among Maasai in Tanzania
Source: PLOS Digit Health. 2023 May 19;2(5):e0000254. doi: 10.1371/journal.pdig.0000254 (PMC10198512; doi:10.1371/journal.pdig.0000254)
Supplement: Attachment — Submitted filename: Ondoilo_Manuscript 2march2022.docx [file pdig.0000254.s002.docx]

“The phone number is telling us good things which we didn’t know before.” Use of Interactive Voice Response Calling for Improving knowledge and uptake of family planning methods among Maasai in Tanzania

Authors: Kennedy Ngowi^1,2^*, Perry Msoka^1^*, Benson Mtesha^1^*, Jacqueline Kwayu^1,2^, Tauta Mappi^1^, Krisanta Kiwango^1^, Ester Kiwelu^1^, Titus Mmasi^3^, Aifello Sichalwe^4^, Benjamin Shayo^2,5,7^, Eusebious Maro^2^ , I. Marion Sumari-de Boer^1,6^

*authors contributed equally

**Affiliations:**

1. Kilimanjaro Clinical Research Institute, Moshi, Tanzania, United Republic of,
2. Kilimanjaro Christian Medical Center, Moshi, Tanzania, United Republic of,
3. The office of Regional Medical office, Arusha, Tanzania, United Republic of,
4. The office of District Medical office, Monduli, Arusha Tanzania, United Republic of,
5. Kilimanjaro Christian Medical University College, Moshi, Tanzania, United Republic of,
6. Amsterdam Institute for Global Health and Development, Amsterdam, The Netherlands
7. Baylor College of Medicine, Houston, TX, USA

**Corresponding author:**

Kennedy M. Ngowi

Kilimanjaro Clinical Research Institute,

Moshi, Tanzania

Phone: +255758048649 Email: k.ngowi@kcri.ac.tz

# Abstract

**Introduction:** Maasai living in Arusha region, Tanzania, face challenges in feeding their children because of decreasing grazing grounds for their cattle. Therefore, they requested birth control methods. Previous studies have shown that lack of knowledge about and poor access to family planning (FP) may be worsening the situation. We developed an interactive voice response calling (IVRC) platform for Maasai and health care workers (HCW) to create a venue for communication about FP to increase knowledge and access to FP. The objective of this study was to explore the effect of the platform on knowledge, access and use of family planning methods.

**Methods.** We enrolled Maasai-couples and HCW in Monduli District (Esilalei ward), Arusha Region, and followed them for 20 months. We applied a participatory action research approach using mixed methods for data collection to develop and pilot an mHealth-platform with IVRC using Maa language. A baseline assessment was done to explore the knowledge about FP. Furthermore, we abstracted information on FP clinic visits. Based on that, we developed the system called Embiotishu. A toll-free number was provided to interact with the system by calling with their phone. The system provides pre-recorded voice messages with information about FP and reproductive health to educate Maasai. The system recorded the number of calls and the type of information accessed. We measured the outcome by (1) a survey investigating the knowledge of contraceptive methods before and after Embiotishu and (2) counting the number of clinic visits (2018-2020) from medical records and feedback from qualitative data for FP used among by Maasai. The acceptability and feasibility were explored through focus group discussions (FGDs) with Maasai and in-depth interviews (IDIs) with HCW.

**Results:** We recruited 76 Maasai couples who were interviewed during the baseline assessment. The overall knowledge on contraceptive increased significantly increased (p<0.005) in both men and women. The number of clinic visits rose from 137 in 2018 to 344 in 2019 and 228 in the first six months of 2020. Implants were the most prescribed family planning method, followed by injections and pills as extracted from medical records. The number of incoming calls, missed calls and questions were 24,033 over 20 months. Out of these calls, 14,547 topics were selected. The most selected topics were modern contraceptives, (mostly implants, condoms, tubal ligation and vasectomy). Natural methods of contraception (vaginal fluid observations, calendar and temperature),

**Conclusion**: Our study has shown that the IVRC system led to an improvement in knowledge about and the access of contraceptives. Furthermore, it has potential to increase access to health information as well as improve dialogue between Health workers and Maasai.

# Introduction

Tanzania has a relatively high fertility rate with about 6.97 children per mother according to the World Health Organization (WHO) [1]. In comparison, globally the rate was 3.0 in 2013 [2]. Limited data is available on fertility rates among Maasai, but data from the year 2000 showed that in Tanzania, Maasai women in the age category 45-49 have a fertility rate of more than 7 and the mean number of children among married women was 6.4 [3]. In 2016, the use of contraceptives and unmet need for family planning among married women in Tanzania was 38.4% and 22% respectively [4]. Despite scarcity of published data, some studies indicate that Maasai are not willing to use family planning methods as they highly value procreation and therefore wish to have a large number of children hence low use of contraceptives and a high rate of unintended pregnancies [5–7]. Consequently, the pastoral community is faced with a dire challenge to provide for their children and families as grazing space has become increasingly limited.

Tanzania has a wide array of health services including reproductive health and family planning services. However, accessibility is low in remote areas especially in the marginalised communities like the Maasai. Furthermore, Maasai may be lacking adequate knowledge about family planning and other reproductive health issues. For instance, Maasai women to become a mother is sign of procreation.[5] Several sexual practices are related to that such as early sexual debut, female circumcision, polygamy and traditional birth outside the hospital [8]. A study among young Maasai women in Kenya showed that the rate of family planning use is low, while there is a high rate of unintended pregnancies [7]. Further, they also found that women think family planning is only used by married women who do not want more children and that family planning methods cause side effects such as infertility and cancer.

Despite the fact that mobile phone coverage in Tanzania is extremely high with >80% and several mHealth programs have been deployed for family planning and reproductive health, the accessibility of such programs pose a challenge to many Maasai who are illiterate and only speak Maa, which is their tribal language [9]. These programs, such as ‘Wazazi Nipendeni’ and ‘Mobile for Reproductive Health’ are in the Kiswahili language and are using SMS (Short Message Service) or text messages [10–12].

Several recommendations have been made including the use of Interactive voice response calling (IVRC) as potential applications to improve the communication and increase knowledge on health-related issues among low-literacy community [13]. Interactive voice response calling (IVRC) makes use of a toll-free number with predefined given information through spoken language and as such could be a way to communicate with Maasai in their own language who often have low literacy and do not quite understand the Swahili language [14].

Based on this, we explored the effect and feasibility of an IVRC system for family planning education among Maasai couples of reproductive age living in Esilalei ward, Monduli District in Arusha Region. The main objective of this study was to explore the change in knowledge about and use of family planning methods after implementation of an educational interactive voice response calling system about family planning and contraceptives for Maasai. Specific objectives were (1) to investigate whether the IVRC system improved knowledge about contraceptives, (2) to examine whether the system increased the access to family planning care, (3) to determine whether the rate of contraceptive use improved and (4) to establish whether the system was acceptable and feasible in use.

# Methodology

## Study design

We conducted participatory action research using mixed methods for data collection. This consisted of a baseline assessment, development of the digital system, deployment of the system, and final assessment among Maasai using several data sources described below. The study was approved by the Kilimanjaro Christian Medical College Research Ethics and Review Committee (CRERC) and the National Health Research Ethics Sub-Committee (NatHREC) of Tanzania.

## Study area and population

Our study was conducted among married Maasai couples, a husband with one of his wives, in Esilalei ward in Monduli District, Arusha Region, Tanzania. We selected Maasai men using convenience sampling. The husband chose one of his wives to participate in the study. The ward consists of three villages which are Esilalei, Losirwa, and Oltukai, and the majority of the population consists of the Maasai tribe. There is one dispensary in the ward that provides family planning services including counselling and provision of some contraceptives methods including oral contraceptive pills, injection, and implants. Other contraceptives methods such as insertion of intrauterine devices and tubal ligation can be accessed at Mto-wa-Mbu Health Centre, about 10 kilometres away from Esilalei. Additionally, health care workers were enrolled for in-dept interview.

## Study procedures

### Baseline assessment

A baseline assessment was done in January and February of the year 2019. Maasai couples were enrolled by firstly screening their eligibility. If eligible, we extensively explained to them about the study and asked for written informed consent to participate. We used the local Maasai language, Maa, for all communication with participants through our Maasai interpreters. If written informed consent was not possible, we collected thumbprints after approving to participate. Consent was given by the husband and wife for being part of the study if they were above 18 years. For men aged below 18 years, we asked consent from their father/elder who is the legally acceptable representative (LAR) in addition to assent from themselves. For women under 18 years, we asked both the husband and the elder to consent and we asked assent from the women. Following written informed consent and oral consent, participants were interviewed using a structured questionnaire to assess knowledge about and use of contraceptives.

Furthermore, we abstracted information on family planning clinic visits from available medical records at Esilalei dispensary and Mto-wa-Mbu health centre to determine the number of clinic visits and prescribed contraceptive methods in the year before implementation of Embiotishu (January 2018-February 2019).

### Development and implementation of Embiotishu

We developed an interactive voice response calling (IVRC) system called Embiotishu. The system was accessible for Maasai by calling a toll-free number. The Embiotishu (which means health in Maa) system was developed based on the information obtained during the basic assessment with guidance of Tanzanian guidelines for family planning. Furthermore, several meetings were conducted with Maasai couples, local health care providers, and study consultant obstetrician gynaecologists, to set the criteria, identify the knowledge gap in Reproductive health and contents of the system. Texts were translated to Maa and categorized based on different themes including reproductive health, family planning, and STIs/HIV/AIDS. We created audio messages by recording the spoken texts in Maa language and loaded them in the Embiotishu system. A menu was developed for the contents and loaded in Embiotishu ***(figure 1).*** Each menu item was assigned a number (e.g. 1,2,3,4 etc) that study participants (Maasai couples) could press on their mobile phone keypad when they wanted to select a certain item. When a study participant calls Embiotishu (s)he hears: “Welcome to Embiotishu, a programme to inform you about reproductive health and family planning. For reproductive health press 1, for information about contraceptives press 2, for information about family planning press 3….(etc)’. Once the IVRC-system recognized the number entered, automatically it routed the request to the particular pre-recorded content. The IVRC-system allows multiple requests from different users in the same call. Before deployment of Embiotishu, we conducted seminars with both parties (Maasai and Health providers) to introduce the system. During the seminars, role plays were used to instruct users about the system, and the toll-free number was provided. We deployed the system for 20 months.

***Figure 1: IVRC content flow***

### Final assessment

After twenty months of follow-up, we repeated the survey to investigate the knowledge of family planning methods among the study participants. We also continued the medical abstraction to explore the trend of family planning utilization in the clinic (access to care) since deploying Embiotishu (March 2019-July 2020). We conducted focus group discussions using semi-structured topic guides with Maasai couples to investigate the acceptability and feasibility of Embiotishu and to explore knowledge and use of FP. In order to investigate the number of times the system has been used and which topic was mostly accessed, we explored the number of incoming calls to Embiotishu and the number of chosen menu items.

## Data collection tools

### Survey with Maasai

A semi-structured questionnaire was administered through face to face interviews during the baseline and final assessment among Maasai couples. Swahili or Maa language was used based on the preference of the interviewee. The questionnaire contained questions on socio-demographic characteristics, marital status, family composition, reproductive history, knowledge about specific contraceptive methods and use of contraceptives. In the final assessment, we added questions to explore their experience with Embiotishu system. Data was collected on a paper form and entered in the REDcap database and stored on the local server.[15]

### Medical records abstraction

A medical records abstraction form was developed based on the standard clinic card for family planning (Reproductive Child and Health card no.5). Data was collected from medical files of the family planning clinics of Esilalei Dispensary and Mto-wa-Mbu Health Centre. The following information was collected; clinic visit date, reproductive history, current contraceptive use and prescribed contraceptives. Data was collected in paper form and entered by trained staff in the REDCAP database directly and stored on a local server.

### FGD with Maasai couples

Focus group discussions were done in the final assessment using a topic guide by a trained qualitative researcher using the Maasai project leaders as interpreters. The topics discussed were: (1) knowledge about the Embiotishu system, (2) “How Embiotishu changed Maasai life”, (3) knowledge about and use of family planning methods, (4) knowledge about the reproductive system, (5) knowledge about sexually transmitted diseases and (6) suggested improvements for the Embiotishu system. Discussions were done in the Maasai language with continuous translation and interaction between the researcher and interpreters in separate groups of men and women.

### IDI with Health Care Providers

In-Depth interviews were done with Health Care providers during the baseline to understand and obtaining their views on how the Maasai know, access and use contraceptives in order to contribute into the development of Embiotishu system. Later IDIs were conducted during the final assessment to explore their view on the knowledge of family planning method among Maasai people after Embiotishu. Semi-structured interview guides were used assessing (1) knowledge about contraceptives among Maasai, (2) access to family planning methods, (3) improved use of contraceptives because of Embiotishu and (4) the acceptability of Embiotishu system.

### Embiotishu System data

Incoming calls and responses (menu items) data of Embiotishu were obtained automatically via an online dashboard. To access the information, login credentials such as password and username were provided to authorized users. The information recorded included: caller ID, information type, time, date, and call- frequencies. This information was extracted from the system in the form of graphs.

To gain feedback from Maasai couples about Embiotishu, interviews with semi structured questionnaires were conducted during the final assessment among couples to gain their experience on using the Embiotishu. The questions included their general experience about the system, preferred topic, challenges and recommendations to improve the Embiotishu system.

## Data analysis

For quantitative data, we conducted descriptive analyses with SPSS v.27 to investigate changes and trends in knowledge and use. Data from the Embiotishu was displayed in graphs using an excel spreadsheet program to create frequency tables.

Qualitative data were transcribed and translated into English. Transcripts were read and reread by PM, TM, and MS and imported into MaxQDA 2020. We created memos based on the transcripts from where we identified codes for organizing our qualitative data. We conducted thematic framework analyse (PM) with an inductive approach and used the coded transcripts to obtain different themes and subthemes. The themes were related to knowledge, access, and use of family planning methods and the acceptability and feasibility of the Embiotishu system.

To answer objective one on knowledge, we investigated with data from the survey whether the percentage of participants knowing about different contraceptive methods (‘yes’-answers) changed before and after using Embiotishu by conducting McNemar tests. In addition, to get an overall score on the knowledge we summed knowledge about all contraceptives (‘yes’-answers being 1 point) into one score. T-tests were conducted to investigate differences in scores before and after the deployment of the system for both men and women. Furthermore, we analysed data from the final assessment FGD and IDI through identifying themes related to knowledge about family planning methods.

To answer the second objective on access to family planning methods, we examined whether the number of clinic visits for family planning between baseline and final assessment increased. Descriptive analysis was used to describe the percentage of the data from the survey.

To answer the third objective on the use of family planning methods, we investigated the difference in use of contraceptives between baseline and final assessment with a Chi-square test. In addition, we identified themes related to the use of family planning methods discussed in the FGD.

To answer the fourth objective on acceptability and feasibility of Embiotishu, we identified themes related to these topics from both the FGD and IDI. In addition, we descriptively analysed data about the experience with the system from selected topics recorded by the system and the survey in the final assessment.

# Results

## Study population

We recruited 76 Maasai couples who were interviewed during the baseline assessment. The mean age was 25[SD:5.9] years for women and 32.5[SD:10.5] for men. Of women, 65% had no formal education and 27% completed primary education while 35% among men had no formal education and 47% completed primary education. Also, 63% and 68% of women and men respectively were Christian. Among women, no one was employed, while 9% of men were employed. Other men were engaged in farming activities for income. ***(Table 1)***

Four FGD were held with 10 to 13 Maasai in each group. Two groups were Maasai women and the other two were Maasai men. We also conducted four IDI with HCP.

Table 1: Socio-demographic characteristics of Maasai

| Characteristics | Women | | Men | |
| --- | --- | --- | --- | --- |
|  | Number | Percent, % | Number | Percent,% |
| Total | 76 | 100% | 76 | 100% |
| Age (years)  Mean [SD] | 25[5.9] |  | 33 [10.1] |  |
| Level of education  No formal education  Some primary education  Primary education  Secondary education  Higher education  Missing | 50  4  21  1  1  1 | 65%  4%  27%  1%  1%  1% | 27  8  34  6  0  1 | 36%  10%  45%  8%  0%  1% |
| Religion  Christian  Muslim  No religion  Other religion | 47  0  29  0 | 62%  0%  38%  0% | 52  0  22  0  2 | 68%  0%  29%  0%  3% |
| Literacy rate (can read Swahili) | 27 | 36% | 48 | 63% |
| Own a cell phone | 31 | 41% | 60 | 79% |
| Employed | 0 | 0% | 7 | 9% |
| Husband has More than one | 31 | 41% | 33 | 43% |
| Wife Included in the study (1^st^ 2^nd^ 3^rd^ )  1^st^ wife  2^nd^ wife  3^rd^ wife and more | 15  19  10 | 20%  25%  12% | -  -  - | -  -  - |
| Age started to live with a partner  Mean [SD] | 17 [5.9] |  | 23[4.8] |  |
| Age when having had 1^st^ child  Mean [SD] | 18 [4.1] |  | 27[5.8] |  |

## Knowledge about family planning and contraceptives

Table 2 describes the difference in knowledge about contraceptives before and after implementation of Embiotishu among women and men. For women, knowledge about all contraceptives significantly increased (p<0.05). The highest increase was for knowledge about intra-uterine device (IUD), condoms, and Lactational Amenorrhea Method. For men it only increased for vasectomy, injection, implants, contraceptive pills, condom, and withdrawal (p<0.05)

Table 2: Knowledge about contraceptives

| **Knowing about contraceptive** | **Women** | | | **Men** | | |
| --- | --- | --- | --- | --- | --- | --- |
|  | Basic assessment  n(%) | Final assessment  n(%) | P-value | Basic  Assessment  n(%) | Final  Assessment  n(%) | P-value |
| Tubal ligation | 24(31.6) | 55(72.4) | <0.001 | 41(53.9) | 42(55.3) | 0.6 |
| Vasectomy | 10(13.2) | 47(61.8) | <0.001 | 12(15.8) | 23(30.3) | 0.03 |
| Loop/intra-uterine device (IUD) | 27(35.5) | 64(84.2) | <0.001 | 58(76.3) | 63(82.9) | 0.013 |
| Injection | 45(59.2) | 69(90.8) | <0.001 | 57(75.0) | 66(86.8) | <0.001 |
| Implants | 50(65.8) | 69(90.8) | <0.001 | 60(78.9) | 66(86.8) | <0.001 |
| Oral contraceptive pills | 44(57.9) | 65(85.5) | <0.001 | 50(65.8) | 62(81.6) | <0.001 |
| Condom | 23(33.3) | 59(77.6) | <0.001 | 54(71.1) | 65(85.5) | <0.001 |
| Emergency contraceptives (morning-after-pill) | 25(32.9) | 59(77.6) | <0.001 | 38(50.0) | 41(53.9) | 0.3 |
| Cycle beads to count the days | 8(10.5) | 57(75) | <0.001 | 21(27.6) | 27(35.5) | 0.2 |
| Lactational Amenorrhea Method (LAM) | 30(39.5) | 62(81.6) | <0.001 | 35(46.1) | 24(31.6) | 0.18 |
| Calendar rhythm method | 51(67.1) | 62(81.6) | 0.003 | 71(93.4) | 66(86.8) | 0.1 |
| Withdrawal method | 52(68.4) | 64(84.2) | <0.001 | 56(73.7) | 67(88.2) | 0.001 |

The overall knowledge on contraceptive use between men and women before and after implementation of Embiotishu both increased significantly (P-value<0.001). ***(table 3)***

Table 3: Difference in knowledge about all contraceptive methods (sum score) before and after Embiotishu

| **Gender** | **Basic assessment**  **mean(SD) – Sum of all contraceptive methods** | **Final assessment**  **mean(SD) – Sum of all contraceptive methods** | **P-value** |
| --- | --- | --- | --- |
| **Women** | 5.0(3.2) | 10.6(2.3) | <0.001 |
| **Men** | 7.5(2.8) | 9.0(1.8) | <0.001 |

The findings from qualitative data indicated that the Embiotishu system increases the knowledge about FP by Maasai. As a result, it positively influences their attitude towards practicing family planning. This was witnessed when they stated that:

***“We have seen the use of Embiotishu numbers is clearer and easier to understand as it teaches us about family planning, so now we know family planning and many other things” (Male, 28 years)***

The information about family planning methods in the Embiotishu system led to enable Maasai couples to make a conscious effort to limit or space the number of children they have and reduce fertility. It was evidenced by:

“***We have heard a lot of good things in the Embiotishu system which in general are focusing on putting us in the position of being able to plan several children in our families.” (Female, 23 years)***

Family planning gives knowledge of the reproductive systems and advances the health of women and children by lowering the proportion of pregnancies that could be high risk and reducing the number of unplanned pregnancies and births. One was aware of family planning benefits and was quoted as.

***“And the other thing you get from it, is to know the parts of Male and Female reproductive systems, to understand that you can stay for how long or some days without having sex with your wife. Such things are described, which gives us more education.” (Male, 35 years)***

Another respondent said:

***“And the things that are being discussed, for real, if someone decides to consider it will help you because it tells you a lot of things. Particularly about the female and male reproductive system, and also to understand the menstrual cycle of your wife.” (Male, 40 years)***

From the FGDs, we found that the knowledge from the system among Maasai people accomplishes what they required and make them to be able to plan well their families. In previous days, they were staying far from their wives worrying to have a greater number of children which they haven’t planned yet. This was reported as:

***“The calendar method is good because it has no hormones and it will show you that she has cheated because she will get pregnant.” (Male, 55years)***

## Access to family planning care

In the year before the implementation of Embiotishu (2018), the number of visits for family planning methods was lower compared to the years after implementation (2019 and 2020). ***(figure 2)***

|  | Total |
| --- | --- |
| 2018 Qtr1 | 22 |
| 2018 Qtr2 | 34 |
| 2018 Qtr3 | 25 |
| 2018 Qtr4 | 54 |
| 2019 Qtr1 | 88 |
| 2019 Qtr2 | 83 |
| 2019 Qtr3 | 43 |
| 2019 Qtr4 | 114 |
| 2020 | 214 |

***Figure 2****: Number of family planning clinic visits per quarter per year*

Findings from qualitative data show that the Embiotishu system increased the perception concerning family planning. It increases the number of clinic visits. Also, family planning provides benefits to the families and their personal development. One of the nurses we interviewed told us:

***“Yes, the system made changes in our work, because customers seem to understand well the Family Planning system through the details they already got.” (Nurse, 44 years)***

Another nurse added and said:

**“*These days, men around here realize the importance of family planning. Some even accompany their wives to the clinic.” (Nurse, 43 years)***

Education introduced among Maasai people concerning family planning increased awareness on the use of contraceptives, and increased the availability. It is an essential factor to fertility decline. One of the participants told:

*“****So, if people get awareness about using contraceptives like paracetamol, if you feel pain you can send a child to buy it at the shop it will be easy as you can select several points to put boxes of condoms and anybody who want it may go there and pick it.”*** ***(Male, 55years)***

## Use of family planning methods

Out of the 76 couples who were interviewed before the implementation, only 26(27%) couples revealed to use contraceptive methods before Embiotishu. After implementation, 69(73%) couples revealed to use contraceptive with P-value<0.001.

Before and After implementation of Embiotishu, from the medical records, we saw that implant was the most prescribed family planning method from 24(3%) before to 447 (65%), followed by injection from 9(1%) to 105 (15%), condoms from zero to 47(7%) and contraceptive pills from zero to 36 (5%). ***(figure 3)***

|  | Before | After |
| --- | --- | --- |
| Condom | 0 | 48 |
| Implants | 24 | 455 |
| Implants+Condom | 0 | 3 |
| Injection | 9 | 102 |
| IUD | 6 | 9 |
| Pills | 2 | 43 |
| Pills+Condom | 0 | 1 |

*Figure 3: Number of contraceptives prescribed from medical abstraction*

Qualitative data showed that informing Maasai families about FP was associated with acceptance of family planning in the context of reducing family size and ability to use contraceptives. The majority of the Maasai indicated that after being educated their perception has increased and able to handle well their families. This was supported:

***“Yes, it is true people have changed. For example to my side up to this stage am now, we are thinking that if I have five children and they need to go to school so it makes us to think about spacing to be able to take care of these we have already. So, it is nice and it is making people to change their perception.” (Male, 36 years)***

The acceptability of contraceptives was also accompanied by the possible choice of natural contraceptive, which also proved to be beneficial. The woman commented and said:

***“The calendar method is good because it has no hormones and it will show you that she has cheated because she will get pregnant” (Male, 55 years)***

Another woman from the focus group discussion shared:

***“We are no longer giving birth too much because we know family planning. Because through the Embiotishu number, we were told things to do and these things are practical”. (Female, 43 years)***

This is also supported by one of the group members saying that:

***“Contraceptives methods make us not to have unexpected pregnancies” (Female, 18 years)***

A great example of use of family planning methods was mentioned by the following participant:

***“Embiotishu has brought to us many very good things and this is because now a person can dial that number and listen. You know, before we did not have sex with our wives while they are still lactating. If a woman lactated for five years you stay also for those years without having sex with her but right now things have changed, when we go to the market you come with condoms and then you can have sex with her even if she has three or five months after giving birth.” (Male, 25 years)***

This was also explained when we were interviewing nurses. It was reported that:

***“Yes, they tell each other this is good, you can stay with it up to five years, so it is good depending on the number of children they have.” (Nurse, 29 years)***

## Acceptability and feasibility of Embiotishu

### Feedback from Maasai

Out of the participants (N=152) enrolled in the study, 105 were interviewed about the experience with the system; 57(95%) of men and 48(91%) of women. Almost all men and women indicated the system was well understood and it was easy to navigate through the information. Ninety-four percent of women (N=53)and eighty-seven percent of men(N=42) indicated that the system performed well. Nearly all indicated the most interesting topics were reproductive health and contraceptive methods. Furthermore, others recommended topics related to sexual transmitted diseases and infertility to be added in the Embiotishu. Few participants indicated to communicate with health care workers about information from Embiotishu.

During FGD discussions, Maasai described that dry season causes substantial migration of the family and their animal mainly to search for water and food.

As mentioned by one participants

***“When a dry season comes like now, we only concern about where to get green grass and water for our animals and family. We cannot be thinking about family planning issues or access information in Embiotishu because we are struggling here and there in search of water.” (Male, Age 45 years)***

Embiotishu proofed to be of advantage also to the marital relationship. This is when the marital sexual relationship was no longer limited during the lactation period. One participant mentioned:

***“Embiotishu has brought to us many very good things and this is because now a person can dial that number and listen. You know before we did not have sex with our wives while they are still lactating. If a woman lactated for five years you stay also for those years without having sex with her but right now things have changed, when we go to the market you come with condoms and then you can have sex with her even if she has three or five months after giving birth.” (Male,25 years)***

Another respondent shared;

***“ I always call Embiotishu number seven times a day”(Female, 45years)***

The same respondent reported that:

***“I always like to dial Embiotishu number and press one it tells you about your reproductive system in general, so you will know that this is good because it instructs you in a direction that when you follow it you will not fall or it will not let you down.”(Female, 45 years)***

### Access to contraceptive contents in the Embiotishu system

A total number of 24,033 incoming calls including missed calls and questions were recorded by the system in the period of 20 months from 2019 to 2021. Out of those, 14547 topics were selected. The highest number of calls were recorded in March, April, and May of 2020. ***(Figure 4)***

|  | Total |
| --- | --- |
| 2019 Qtr3 Sep | 15 |
| 2019 Qtr4 Oct | 12 |
| 2019 Qtr4 Nov | 12 |
| 2019 Qtr4 Dec | 155 |
| 2020 Qtr1 Jan | 2205 |
| 2020 Qtr1 Feb | 1759 |
| 2020 Qtr1 Mar | 3890 |
| 2020 Qtr2 Apr | 2437 |
| 2020 Qtr2 Jun | 1579 |
| 2020 Qtr2 May | 4505 |
| 2020 Qtr3 Jul | 1248 |
| 2020 Qtr3 Aug | 1681 |
| 2020 Qtr3 Sep | 902 |
| 2020 Qtr4 Oct | 561 |
| 2020 Qtr4 Nov | 508 |
| 2020 Qtr4 Dec | 391 |
| 2021 Qtr1 Jan | 498 |
| 2021 Qtr1 Feb | 507 |
| 2021 Qtr1 Mar | 510 |

*Figure 4: Trend of calls in Embiotishu*

#### Modern Contraceptives

In 400 calls, the topic of hormonal contraceptives was selected. Among those calls, in 25% implant was selected, in 24% copper IUD, in 18% Mirena (hormonal IUD), in 4% pills (oestrogen and progestogen) and 4% minipill (progestogen hormone only) were selected*.* ***(Figure 5)***

|  | Total |
| --- | --- |
| Copper | 95 |
| Implant | 101 |
| Minipill | 16 |
| Mirena | 60 |
| Pill | 16 |
| Emergency contraceptives (morning-after-pill) | 16 |

*Figure 5: access to hormonal contraceptive content in IVRC*

Out of those 197 calls, the barrier methods topic male condoms were selected 65 times (33%) and female condoms 40 times (20%). For permanent methods, tubal ligation was selected 42 times (21%) followed by vasectomy (12%). ***(figure 6)***

|  | Total |
| --- | --- |
| Tubal ligation | 42 |
| Vasectomy | 24 |
| Male Condom | 65 |
| Female condom | 40 |

*Figure 6: non hormonal contraceptives*

#### Natural contraceptives

In a total of 1,376 calls, the topic of natural contraceptives was selected. The most selected natural contraceptive was the observing of vaginal fluid with 441 times (33%) followed by monitoring the women’s temperature with 440 times (32%) and avoiding sex on danger days 391 times (28%). In a few calls, withdrawing the penis before ejaculation and LAM (exclusively breastfeeding) methods were selected. ***(figure 7)***

|  | Total |
| --- | --- |
| Avoiding sex on danger days | 391 |
| Fluid | 441 |
| Lactational Amenorrhea Method (LAM) | 63 |
| Tempature | 440 |
| Withdrawing the penis | 41 |

*Figure 7: Natural contraceptive use*

# Discussion

Our study shows that the system led to an improvement in knowledge about and use of contraceptives, family planning, and sexual and reproductive health. This was directly shown through the survey in which knowledge about the number of family planning methods increased significantly and the use of contraceptive methods increased after our program. Similar results were observed during focus group discussions. Data from the discussions showed that the implementation of the Embiotishu system positively changed the Maasai attitudes towards practicing family planning. Furthermore, participants revealed that the education provided by IVRC, makes them to be able to identify effective contraceptives. Medical abstraction results showed that the trend of visits for family planning increased after the implementation of Embiotishu. It was revealed by nurses that, the Embiotishu changed the Maasai perception concerning family planning which resulted in men and women’s’ understanding the importance of family planning. Therefore, the system has potential in limiting the number of children, spacing between children and reducing fertility.

These results are comparable to a study conducted in Uganda which showed that learning about family planning among women living along lake Victoria, increased their ability to plan for their families.[16] Another study conducted in Kenya describes that knowledge on contraceptive among pastoralists, improves access to family planning and decreased the burden to the household due to the higher cost of living.[17] This was related to the study by Allegretti et al. which showed that pastoral household heads find it difficult to support larger families due to the rising demand for money which does not match with the available cash income sources.[18] Further, this was also stated by Leah et al. who found that access to reproductive health knowledge prevents unwanted pregnancies, especially among adolescents, and also prevents sexually transmitted diseases and AIDS. [19]

A prior study has shown that Maasai men seem to have a negative perception about family planning, because it will lower the birth rate and cause infertility. Also, Maasai believe that having many children was a sign of wealth and prestigious in the community.[20] However, our study shows that Maasai were interested to use family planning and apply child spacing in order to satisfy the family’s needs including education, shelters, and food.

During the discussion, Maasai women expressed to be empowered by Embiotishu particularly on the topics regarding ,family planning which led them to have a strong decision on child spacing. This was also shown in the study from Kenya which showed that education about contraceptives empowered women and makes them more aware of better decisions on contraceptive use.[21] Furthermore, few Maasai selected information about condoms as well as hormonal contraceptives in Embiotishu. As described in the previous studies, the sociocultural context contributes to a negative perception as Maasai believe the use of condoms leads to waste of semen. Also the use of hormonal contraceptives cancer, and increasing weight and killing women’s eggs (infertility).[5,21]

Another finding was the high volume of incoming calls to the system in certain months. It was revealed during the discussion to be strongly related to the rain season. Many areas become green for grazing the cattle and as a result, Maasai men spend more time with their families and wives at bomas. A study in Kenya described the same kind of trend during the rainy season, in which Maasai men tend to spend time on other issues such as traditional ceremonies, selling or buying cattle as well as expanding the families.[20]

## Limitations

The study was conducted in only one village ward and the number of participants was small. This makes the representativeness of the total Maasai population in Tanzania questionable. As Esilalei is situated in a rather accessible area with some basic health care services, we expect that the population is not representative of the total Maasai population who live in the less accessible areas. In addition, we have not conducted an intervention study comparing results between people exposed and not exposed to Embiotishu. We can therefore not conclude that our intervention is effective. Though our study provides enough proof for the intervention to be investigated in a larger trial to determine its effectiveness.

## Strengths

The development of the project was based on a participatory method whereby the Maasai community was involved at the early stages of brainstorming the idea. This helped us to familiarize ourselves with indigenous knowledge, challenges, and barriers which guided us to tailor the IVRC contents to specific targets. In addition, by using a mixed-methods approach and different sources of data we obtained rich data which made us to be able to answer the objectives and to give a good insight into the context of our data.

## Conclusion

Our study has shown that the IVRC system led to an improvement in knowledge about and the use of contraceptives. Embiotishu also appeared to be feasible and acceptable among the Maasai. Furthermore, it has the potential to improve access to health information. However, it is important to consider the norms and culture of the Maasai community on the topics of family planning, reproductive health and contraceptives. Therefore, it is relevant to incorporate all attributes related to the socio-cultural aspects of Maasai to ensure that future interventions are effectively empowering the Maasai community. Furthermore, the rapidly growing migration of Maasai to urban areas in recent years and mobile technology services facilitating sharing information in distance location. Future studies should continue to explore the effect of this digital technology for continuous advocacy of use of contraceptive and family planning among Maasai community in randomized controlled settings.

# Acknowledgments

We would firstly like to thank Voice.global for financially supporting our study under the linking and learning grant for Tanzania. We thank our research assistants from the Maasai community, Mbayani Lemkoko, Esta Mbayani, Elizabeth Morine, Rueben Moitiko who assisted us with collecting all data during interviews and focus group discussions. We also thank our partner in this project which is the African Roots Foundation through Chris Pilley. Further, we also thank our colleagues who were involved in the proposal development and preliminary focus group discussions which were Godfrey Kisigo, Iraseni Swai, and Martha Oshosen. Lastly, we thank the participants for providing us with valuable and sensitive information.

# Reference list

1.	World Health Organization. Global Health Observatory country views: United Republic of Tanzania statistics summary (2002 - present). Vol. 69030. 2018.

2.	Kaneda CH. 2013 World Population Data Sheet [Internet]. Population Reference Bureau. 2013 [cited 2022 Jan 27]. Available from: https://www.prb.org/resources/2013-world-population-data-sheet/#:~:text=OTHER HIGHLIGHTS FROM THE 2013,high of 7.6 in Niger.

3.	Coast E. Maasai Demography. Vol. 2000. London, UK; 2000.

4.	Ministry of Health, Community Development, Gender E and CD es S, Zanzibar M of H, Salaam NB of SD es, Zanzibar O of CGS, ICF, Rockville MU. Demographic and Health Survey and Malaria Indicator Survey 2015-16. Demographic and Health Survey. Dar es Salaam, Tanzania, and Rockville, Maryland, USA; 2016.

5.	Coast E. Wasting semen: Context and condom use among the Maasai. Cult Heal Sex. 2007;9(4):387–401.

6.	TDHS. Tanzania Demorgraphic and Health Survey Indicator Survey (TDHS-MIS) 2015-2016. Dar es Salaam, Tanzania, Rockville, Maryland, USA MoHCDGEC, MoH, NBS, OCGS, ICF. 2016;1(1):1–630.

7.	Stats MA, Hill DR, Ndirias J. Knowledge and misconceptions surrounding family planning among Young Maasai women in Kenya. Glob Public Health [Internet]. 2020;1847–56. Available from: https://doi.org/10.1080/17441692.2020.1788112

8.	Birks LK, Powell CD, Thomas AD, Medard E, Roggeveen Y, Hatfield JM. Promoting health, preserving culture: Adapting RARE in the Maasai context of Northern Tanzania. AIDS Care - Psychol Socio-Medical Asp AIDS/HIV. 2011;23(5):585–92.

9.	TCRA. QUARTERLY COMMUNICATIONS STATISTICS October-December October-December 2019 Operators’ Submissions NOTE: 2019;(December).

10.	Kaufman MR, Harman JJ, Smelyanskaya M, Orkis J, Ainslie R. “Love me, parents!”: Impact evaluation of a national social and behavioral change communication campaign on maternal health outcomes in Tanzania. BMC Pregnancy Childbirth. 2017;17(1):1–10.

11.	mHealth Tanzania. Wazazi Nipendeni: How text messages are supporting mothers in Tanzania. 2013.

12.	FHI360. Mobile for Reproductive Health (m4RH). 2021.

13.	Tripathi V, Arnoff E, Bellows B, Sripad P. Use of interactive voice response technology to address barriers to fistula care in Nigeria and Uganda. mHealth [Internet]. 2020 Apr 5;6:12. Available from: https://pubmed.ncbi.nlm.nih.gov/32270004

14.	Mudliar P, Donner J. Experiencing interactive voice response (IVR) as a participatory medium: The case of CGNet Swara in India. Mob Media Commun [Internet]. 2015 Mar 10;3(3):366–82. Available from: https://doi.org/10.1177/2050157915571591

15.	Harris PA, Taylor R, Thielke R, Payne J, Gonzalez N, Conde JG. Research electronic data capture (REDCap)-A metadata-driven methodology and workflow process for providing translational research informatics support. J Biomed Inform [Internet]. 2009;42(2):377–81. Available from: http://dx.doi.org/10.1016/j.jbi.2008.08.010

16.	Nanvubya A, Wanyenze RK, Nakaweesa T, Mpendo J, Kawoozo B, Matovu F, et al. Correlates of knowledge of family planning among people living in fishing communities of Lake Victoria, Uganda. BMC Public Health [Internet]. 2020;20(1):1642. Available from: https://doi.org/10.1186/s12889-020-09762-7

17.	Nkedianye DK, Ogutu JO, Said MY, Kifugo SC, de Leeuw J, Van Gardingen P, et al. Comparative social demography, livelihood diversification and land tenure among the Maasai of Kenya and Tanzania. Pastoralism [Internet]. 2020;10(1):17. Available from: https://doi.org/10.1186/s13570-020-00165-2

18.	Allegretti A. “BEING MAASAI” IN MARKETS AND TRADE: THE ROLE OF ETHNICITY-BASED INSTITUTIONS IN THE LIVESTOCK MARKET OF NORTHERN TANZANIA. Nomad People [Internet]. 2017 Nov 2;21(1):63–86. Available from: http://www.jstor.org/stable/44652673

19.	Mbugua N. ACCESS TO SEXUAL AND REPRODUCTIVE HEALTH CARE SERVICES BY ADOLESCENT GIRLS AGED 15-19 YEARS AMONG PASTORAL COMMUNITIES IN NAROK COUNTY, KENYA. NJOKI MBUGUA LEAH (BSc. FND) P57/CTY/PT/23679/2011 DEPARTMENT OF COMMUNITY HEALTH. 2015;

20.	Kock L, Prost A. Family Planning and the Samburu: A Qualitative Study Exploring the Thoughts of Men on a Population Health and Environment Programme in Rural Kenya. Vol. 14, International Journal of Environmental Research and Public Health . 2017.

21.	Stats MA, Hill DR, Ndirias J. Knowledge and misconceptions surrounding family planning among Young Maasai women in Kenya. Glob Public Health. 2020 Dec;15(12):1847–56.
