# Supplementary material for: “The phone number tells us good things we didn’t know before.” Use of interactive voice response calling for improving knowledge and uptake of family planning methods among Maasai in Tanzania
Source: PLOS Digit Health. 2023 May 19;2(5):e0000254. doi: 10.1371/journal.pdig.0000254 (PMC10198512; doi:10.1371/journal.pdig.0000254)
Supplement: Attachment — Submitted filename: Response letter Plos digital.docx [file pdig.0000254.s003.docx]

P.O.BOX 2236 Email: kcriadmin@kcri.ac.tz

Academic Centre for Evidence Based Health Interventions

An institution of the Good Samaritan Foundation

Moshi,Tanzania	Website: http://www.kcmc.ac.tz

Telephone: 255-27-2754201	Website: http://www.kcri.ac.tz	Fax: 255 27 2753368	Date: 12/10/2022

Chief Editor	Ref: PDIG-D-22-00061

PLOS Digital Health

Dear Editor,

Firstly, we would like to thank you for the opportunity to revise our paper based on the provided reviewer comments. We very much appreciate the critical and constructive comments shared by the reviewers on this manuscript. The suggestions and inputs are very clear and useful for improving the scientific value of the manuscript. We are resubmitting the revised manuscript with the suggestions incorporated as given by the reviewers. We have provided an extensive explanation and made thorough changes to comply with each comment point-by point. Our responses to all the comments are as follows.

**Reviewer #1:** Dear Authors, this is great work in addressing those individuals as it helps to do further research for future. therefore try to amend the comments given in the source document then proceed accordingly.

**Response**

We thank the reviewer for the positive remarks

**Reviewer #2:** There is no literature review, at least to show the gap between the previous studies & to show what is the new in the current study

**Response**

Thanks for point out this. We agree that this is an important consideration however, we could not find any proper references about previous studies particularly in our settings have shown Maasai using mobile phones to increase the knowledge on family planning methods or contraceptives. Therefore, we referenced this paper(9) which primarily focused on understanding the challenges of reproductive services access and uptake contraceptives among women living in a rural areas in Tanzania.

**Changes**

We added ref [9]

Nyblade L, Stockton M, Nyato D, et al.. Perceived, anticipated and experienced stigma: exploring manifestations and implications for young people's sexual and reproductive health and access to care in north-western Tanzania. Cult Health Sex 2017;19:1092–107. 10.1080/13691058.2017.1293844

**Comment**

Limitation & strength should be moved to conclusion and the title should be conclusion & Future work

**Response**

We appreciate the reviewer’s comment. In the revised version, we have moved the strength to conclusion and include the heading name “ conclusion & Future work” as per reviewers recommendation. However, since limitation contains important specific information which describes the constraints of our study findings based on based on applied methods, we therefore believe the limitation should not include in the suggested paragraph.

**Comment**
Methodology should be reformulated, to many speech which need to be minimize
It seems that it’s a copy from PhD or MSc thesis , so it’s need to be formulated

**Response**

We appreciate the reviewers comment. However due to the fact that this is original work, we respectfully disagree that the methodology was copied from thesis. Though that we have tried to shorten on a number of aspects, while we also believe that a mixed-methods study using a number of data sources requires more explanation of methods. In addition, we involved an English-speaking native, so we hope it now matches the journal standard.

Reviewer #3: This research article shows how digital health solutions can be applied to increase knowledge and improve health care in rural and undersupplied areas. Furthermore, the authors found a smart solution in the Interactive Voice Response (IVR) system to overcome literacy barriers. The different methodologies and outcome sources used in implementing and evaluating the IVR system raise the quality of this research.
Nevertheless, there are some concerns regarding formatting and language, completeness of the methods, data availability, figures, precision regarding the results and missing discussion points. These are described in the following.

**Comment**

Formatting and language: The formatting of the text is unclean in many places. There are double spaces, misplaced commas and dots, unfinished phrases (e.g. Abstract, last phrase of the results), and citation outside the phrase in the manuscript. Moreover, the manuscript should be revised concerning language and grammar before publication.

**Response:**

We thank the reviewer for pointing this out. We have involved a native-English speaker for proof-reading and grammar correction. Corrections on grammar and sentence construction can be found throughout the revised paper.

**Methods:**

**Comment**
Please explain the study design (participatory action research using mixed methods) more deeply for non-experts in this field.

**Response**

We appreciate the reviewer’s comment. We have carefully tried our best to provide an additional information on how we performed the participatory method. Foristance, how we interacted with participants and key stakeholders during design and development of the IVR system. In addition, we have revised the previous texts and provided non-expert language to elaborate the meaning of the mixed method design in page **5** paragraph 1

**Comment**
A statement regarding the Declaration of Helsinki is missing.

**Response**

We have added the information accordingly page **5** paragraph **2**

**Comment**

How were health care workers selected for the study? How many were included? Are nurses and health care workers the same participant group?

**Response**

We thank the reviewer for this comment. In the revised manuscript, we have added a statement describing how the health care workers were selected at both baseline and finial assessment (page **8** paragraph **4**). Also, we regret not having the number of the nurses included, this has been corrected in (page **19** paragraph **1)**. In the context participants groups, all personnel involved in the family planning services were rereferred as HCW similarly to definition found in the National Health Operational Guidelines of Tanzania.

**Comment**
In the study procedures it is stated that Maasai couples were screened for their eligibility. What are the eligibility criteria?

**Response**

We have added information (page **5** paragraph **4)** to clarify the inclusion criteria in the revised version according to the suggestions

**Comment**
Add a protocol of the semi-structured questionnaire to the supporting information or describe the relevant questions that were used for data analysis.

**Response**

We greatly appreciate the Reviewer’s insightful comments. Since the questionnaire contains many questions, In the revised manuscript, we have added it as the supporting information (Annex).

**Comment**
Was the medical records abstraction collected for the population in general or specifically for the participants?

**Response**

We appreciate the reviewer’s comment and regret not having discussed this clearly in the previous version. In need, the medical records abstraction were collected for the general population. We have described the information in the revised version page **6**, paragraph **1**.

**Comment**
Explain or cite thematic framework analysis.

**Response**

As suggested by the reviewer, we have added the reference citing the information regarding the thematic framework analysis. The study referred about theme development in qualitative content analysis and thematic analysis.

**Changes:**

We referenced this paper[ref no 16] Vaismoradi M, Jones J, Turunen H, Snelgrove S. Theme development in qualitative content analysis and thematic analysis. J Nurs Educ Pract. 2016;6(5).

**Comment**
Study course: The study course and duration of study phases (deployment and development of the IVR, assessment time points) is not clearly defined. A figure could be helpful here.

**Response**

We agree, and the figure have been added in the revised version in page 8

**Comment**
Data availability: Data is not available yet.

**Response**

We have clarified this that data will be available after agreement have been signed. This is according to the National IRB

**Figures:**

**Comment**
Figure 1: Please explain the figure in the legend and in the text. What do arrows mean?

**Response**

This details are now included in the revised manuscript based on reviewers comment

**Comment**

Difference between dashed and solid line? What do numbers mean? How does the IVR work?
 All figures and tables: Please explain figures and tables in the respective legend.

**Response**

These have been revised and included in the revision as suggested.

**Results:**

**Comment**
It is not clear which data from which source was used for which analysis. E.g., family planning methods: How did couples revealed to use contraceptives? Which question was asked?

**Response:**

The source material used to collect data have been included as supplementary materials in the revision.

**Comment**
Table 1: What do the numbers in the religion cell mean? Was there no information available for the remaining percentage?

**Response**

This section has been deleted since the numbers and percentage were not used in analysis descriptions.

**Comment**
Table 1: Not all participants owned a cell phone. How did those without a cell phone take part in the study?

**Response**

All Participants who did not own a cell phone during baseline were provided with one before participating in the IVR.

**Comment**
Table 2: How was the knowledge assessed exactly? Which questions were asked?

**Response:**

In need we used questions from the DHS which to assess the knowledge. We have added the questionnaire in the Annex 1.

**Comment**
Table 2: It looks like there is a difference between women and men, regarding the knowledge at baseline and the increase in knowledge from baseline to final assessment. Could be an interesting topic for the discussion. Did women used the IVR system more and accepted it better?

**Response**

We are grateful for this comment as it points to an important rationale between women and Men.

We have added the suggested content to the revised manuscript on page **22** paragraph **4** accordingly**.** We described the feedback from women regarding the level of intervention satisfaction, with majority of women expressing that the system was easy to understand.

**Comment**
Table 2: For men, there are some cells where the knowledge decreased. How is that possible? Do you have an explanation for that?

**Response**

We have clarified in the manuscript that traditionally, during the dry season Maasai men were mostly away with the cattle, which had resulted to lower participation in study activities (page **24** paragraph **2**).

**Comment**

Table 3: Please explain how the values are composed.

**Response**

We thanks the reviewers for point this. We have added the annex 3 to describe the composition of the values

**Comment**
Qualitative analysis: The presentation of the results of the qualitative analysis are not sufficient. Which themes and subthemes (according to Data analysis section) were identified? How many statements were allocated to which themes? Please give the reader more information here

**Response**

We very much appreciate this helpful comment and agree that the presentation of qualitative was not clear. In the revised version, we have clarified the number of themes which were deducted from the transcripts as well as provided additional information as Annex 2 describing the definitions and examples if the themes.(page **11**, paragraph **1**)

**Comment**
Access to family planning methods: The number of clinical visits is only descriptively increasing. Can this also be statistically proven?

**Response**

We greatly appreciate for the valuable suggestion from reviewer. However, since this was pilot study to evaluate the feasibility of Embiotishu, therefore we only focused on descriptive statistics to summarize the characteristics of a data set at baseline and final assessment before and after implementation of Embiotishu.

**Comment**
Page 18 first statement: From a woman or a men? Contradictory information here.

**Response**

We regret for this contradiction. The sentence has been revised accordingly

**Comment**
Acceptability and feasibility: Please indicate exact numbers or percentages here. Almost or nearly all is not precise enough.

**Response**

Thank you for the suggestion. We have re-written this part according to the Reviewer’s comments. We have added the numbers and percentage to describe the acceptability and feasibility features

**Comment**
Access to contraceptive contents: What is a missed call? A call where no topic was selected?

**Response**

We thank the reviewer for pointing out this, and to be clearer and in accordance with the reviewer concerns, we have added a brief description as follows: missed calls “no topic where selected”(page **20** paragraph **2**).

**Comment**
A figure where the distribution of selected topics or categories among the calls is depicted could be eventually helpful for the reader.

Response:

This has been added in revised Manuscript as Annex 4

**Comment**
Page 21: “Out of those 197 calls” To what are you referring here? Were there 197 calls about non hormonal contraceptives?

**Response**

Thank you for the suggestion. We have re-written this part according to the Reviewer’s comments. We have added the following : Out of those 197 calls about non hormonal contraceptives.

**Comment**
Is there a relationship between selected topics during IVR calls and change in knowledge/use of contraceptives? If so, the change in knowledge/behaviour could be addressed more clearly to the intervention.

**Response**
We thank the reviewer for the very interesting comment. In fact, the results of our qualitative data illustrate that the topics about FP was highly accepted by Maasai women and provided knowledge in the context of reducing family size and ability to use contraceptives. We have described this in page 23 paragraph 1

**Discussion:**

**Comment**
The study does not directly show that the system leads to the change in outcome variables because there is no control group applied. Please adjust the wording.

**Response**

Thank you for the suggestion. We have re-written this part according to the Reviewer’s

comment. We have therefore changed the first line of the discussion and added that our study found that the intervention was feasible and acceptable. Page 23 paragraph 1

**Comment**
Why has the use of implants increased so much compared to other contraceptives? This could be an interesting topic for the discussion.

**Response**

Considering the valuable Reviewer’s suggestion, we have added new information in the discussion about the high demand of implant contraceptives among women were based on the content of the system. In addition, other reason including that Women experience pressure to have many children as such through the system, they found implant better solution and they don’t have to come back to the clinic too often. We also add relevant citation of the study conducted in Kenya.

**Comment**
-How do you explain the difference between most frequently used contraceptives (hormonal contraceptives, condoms) and most frequently selected IVR topics (natural contraceptives)?

**Response**

We have addressed in the revised version that the most frequently used contraceptives was based on the medical records information that knowledge about hormonal contraceptives and condoms did exist already, but that the study was able to make awareness about availability and safety of such methods. On the other hand, that most frequency selected topic was based on the number of chosen menu in which still be low and therefore they wished to learn more on. It could be.

We hope that our responses have appropriately addressed the editor’s and reviewers’ comments and that the revised paper is now acceptable for publication. We thank you again for the opportunity to revise the paper and your consideration to publish our paper.

Sincerely,

Kennedy Ngowi
