# Supplementary material for: “The phone number tells us good things we didn’t know before.” Use of interactive voice response calling for improving knowledge and uptake of family planning methods among Maasai in Tanzania
Source: PLOS Digit Health. 2023 May 19;2(5):e0000254. doi: 10.1371/journal.pdig.0000254 (PMC10198512; doi:10.1371/journal.pdig.0000254)
Supplement: Attachment — Submitted filename: Responseto comment 10Feb.docx [file pdig.0000254.s004.docx]

P.O.BOX 2236 Email: kcriadmin@kcri.ac.tz

Academic Centre for Evidence Based Health Interventions

An institution of the Good Samaritan Foundation

Moshi,Tanzania	Website: http://www.kcmc.ac.tz

Telephone: 255-27-2754201	Website: http://www.kcri.ac.tz	Fax: 255 27 2753368	Date: 2/10/2023

Chief Editor	Ref: PDIG-D-22-00061

PLOS Digital Health

Dear Editor,

Firstly, we would like to thank you for another opportunity to revise our paper based on the provided reviewer comments. We are again appreciating for additional minor comments shared by the reviewers on this manuscript. The comments were vital for improving the scientific quality of the manuscript. We are resubmitting the revised manuscript with the response to the comments as shared by the reviewers. Our responses to all the comments point-by point are as follows**.**

**Comments to the Author**
6. Review Comments to the Author
Please use the space provided to explain your answers to the questions above. You may also include additional comments for the author, including concerns about dual publication, research ethics, or publication ethics. (Please upload your review as an attachment if it exceeds 20,000 characters)
**Reviewer #1:** they tried to incorporate the comments but still let they modify the English, other comments are just minor that could me modified and I just highlighted my concern in the document too. Thanks

**Response:** We thank the reviewer for pointing this out. Indeed, we have involved a	someone with full professional proficiency in English for proofreading and grammar	correction.

**These corrections have been highlighted in the revised manuscript.**
**Reviewer #3:** Dear author,
considering the reviewers comments, your manuscript has improved substantially, congratulations on that. However, not all comments were implemented properly. There are still some minor issues.

**Response:** We thank the reviewer for the positive remarks that our manuscript has	improved substantially

**General:**
- Formatting: The formatting of the text is still faulty in many places. There are double spaces, misplaced commas and dots, unfinished phrases (e.g. Abstract), and citations outside the phrase in the manuscript. Please review the manuscript carefully
**Abstract:**

**Comment**:
- double use of "increased" in Results section

**Response:** Thank you for point out this. We have corrected this part according to the	Reviewer’s comments

**Comment:**
- last sentence in Results section incomplete

**Response:** Thanks for your review. According to your comment, we have corrected this	mistake in the revised manuscript.

**Methods:**

**Comment:**
- Second sentence grammatically wrong "This was serial design consisted of a baseline	assessment ..."

**Response:** We have corrected the sentence in the revised manuscript.

**Comment:**
- Please add the information that participants without a cellphone were provided with one	to the manuscript.

**Response**: We thank the reviewer for the suggestion. And we agree with the reviewer.	The sentence has been added in the revived manuscript.

**Results:**

**Comment:**
- Page 15: The final quote on that page was already used on page 14, but with a different	reference.

**Response**: We thank the reviewer for pointing out this. In the revised manuscript, the	quote has been removed.

**Discussion:**

**Comment:**
- Page 20 first sentence: that or which

**Response:** Thanks for pointing out this. In need this was typo and we have corrected by	removing the “which” in the revised manuscript

We hope that our responses have appropriately addressed the editor’s and reviewers’ comments

and that the revised paper is now acceptable for publication. We thank you again for the

opportunity to revise the paper and your consideration to publish our paper.

Sincerely,

Kennedy Ngowi
